# *Agaricus sinodeliciosus* and *Coprinus comatus* Improve Soil Fertility and Microbial Community Structure

**DOI:** 10.3390/jof11120866

**Published:** 2025-12-07

**Authors:** Xinxia Lv, Hengsheng Wang, Wenying Wang

**Affiliations:** 1 College of Geographical Sciences, Qinghai Normal University, Xining 810016, China; lvxx1319@163.com; 2 Department of Horticulture and Landscape, Anqing Vocational and Technical College, Anqing 246003, China; 3 School of Biological and Food Engineering, Hefei Normal University, Hefei 230601, China; wang_h_s@126.com; 4 Plateau Science and Sustainable Development Research Institute, Xining 810016, China; 5 Key Laboratory of Biodiversity Formation Mechanism and Comprehensive Utilization in the Qinghai Tibet Plateau, Xining 810016, China

**Keywords:** metagenomic sequencing, macrofungi, bacteria, fungi, physicochemical properties

## Abstract

*Agaricus sinodeliciosus* (*A. sinodeliciosus*) and *Coprinus comatus* (*C. comatus*) are precious macrofungi found in Qinghai Province, China. As decomposers, they play a crucial role in the terrestrial ecosystem. The article takes *A. sinodeliciosus* and *C. comatus* growing in the saline-alkali land of the Qaidam Basin in Qinghai Province as the research objects, and deeply analyzes the influence of the two macrofungi on soil. The results show that, compared with the control soil, the total carbon (TC) content in the soil of *A. sinodeliciosus* and *C. comatus* increased by 27.48% and 113.24%, the total nitrogen (TN) content increased by 95.16% and 108.06%, the hydrolyzable nitrogen (HN) increased by 87.36% and 97.90%, and the available potassium (AK) increased by 182.72% and 596.09%, respectively. In addition, *C. comatus* significantly increased the available phosphorus (AP) by 163.14%. This proves that both macrofungi can enhance soil fertility, and *C. comatus* has a stronger fertilization effect. In terms of soil microorganisms, *A. sinodeliciosus* significantly influenced the distribution of soil bacteria and fungi, increasing the abundance of *Streptomyces* and reducing alpha diversity. *C. comatus* had a greater impact on bacteria, significantly increasing the relative abundance of *Pseudomonas* in the soil, but had no significant effect on fungi. Additionally, there was a close relationship between soil microbial abundance and physicochemical properties. pH, AP, TC, and AK were the main factors influencing bacteria, while total salt was the main factor affecting fungi. These findings reveal that *A. sinodeliciosus* and *C. comatus* influence the soil microenvironment by regulating soil physicochemical properties and microbial communities.

## 1. Introduction

Qinghai Province is located in western China and occupies the northeastern part of the Qinghai–Tibet Plateau, the roof of the world. It has unique natural environment and climatic conditions and has nurtured a large number of precious wild edible fungi [1]. In recent years, the research on wild edible fungi in the Qinghai–Tibet Plateau has been increasing. Through field sampling, morphological observation, molecular biology and other technical means, the species of wild edible fungi in the area were accurately identified and classified, further clarifying the species diversity of wild edible fungi on the Qinghai–Tibet Plateau. At present, the main edible fungi that have been studied more include *Floccularia luteovirens* [2,3,4] and *Morchella esculenta*. There are relatively few studies on *Agaricus sinodeliciosus* and *Coprinus comatus* on the Tibetan plateau. *A. sinodeliciosus*, called “Da fei gu” in China, is a rare wild edible mushroom, growings naturally in mild saline-alkali soil of the Qaidam Basin in Qinghai Province and in parts of Xinjiang [5]. It usually grows 15–20 cm underground. The fruiting body is large and the diameter of the cap is usually 6–20 cm, and the fungus flesh is white, thick and dense, and tastes delicious [6]. *C. comatus*, also known as chicken drumstick mushroom [7], is an edible mushroom species commonly found all over the world [8], and has been artificially cultivated. However, there are still challenges in the artificial cultivation of *A. sinodeliciosus*. The fundamental reason is that the relationship between the wild environment and edible fungi is not well understood.

Soil is the substrate for the growth of macrofungi, providing nutrients for their growth [9]. At the same time, in the process of growth, macrofungi will have a certain impact on soil [10], such as maintaining soil structure, affecting soil properties and soil microbial community [11,12]. Studies have shown that macrofungi cultivation contributes to nutrient bioavailability, and also promotes the degradation of pollutants and the formation of soil aggregates [13,14], affecting soil structure and soil properties. In terms of soil microorganisms, studies have found that macrofungi tend to select specific members of soil microbial communities, thus creating a unique ecological nich [15], to promote the growth of macrofungi. In addition, Zhang C et al. found that the soil microbial communities of *Morchella sextelata* at different growth stages were significantly different [16]. Therefore, it can be found that macrofungi will reshape the soil microbial community for their own growth needs during the growth process [17].

This study took *A. sinodeliciosus* and *C. comatus* growing in the saline-alkali soil of the Qaidam Basin in Qinghai Province as the research objects. By using physical and chemical analysis and metagenomic sequencing methods, it deeply analyzed the influence of the two macrofungi on their surrounding soil environment. The research aims to help further understand the impact of macrofungi on the ecosystem and also provide certain references for the study of the mechanisms by which the two macrofungi adapt to the saline-alkali environment.

## 2. Materials and Methods

### 2.1. Materials

**Figure 1:**
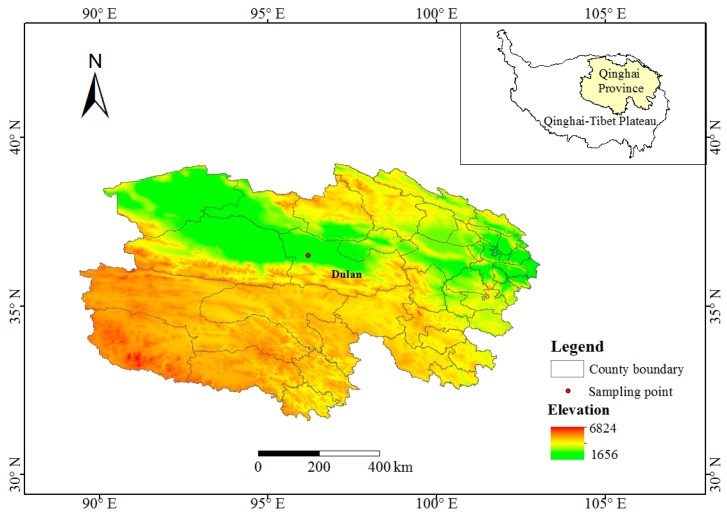
Location of sampling point and altitude of Qinghai Province.

The samples of *A. sinodeliciosus* and *C. comatus* were collected from Nomo Hong Nature Reserve, Zongjia Town, Dulan County, Haixi Mongolian and Tibetan Autonomous Prefecture, Qinghai Province, China (96°11′18″ E, 36°29′54″ N) (Figure 1). This site is located within the Qaidam Basin, the largest basin on the Qinghai–Tibet Plateau [18]. The Qaidam Basin is surrounded by the Kunlun Mountains, the Altun Mountains and the Qilian Mountains [19], with high altitudes around and a lower altitude in the middle (Figure 1, The elevation data were obtained from the Geospatial Data Cloud (http://www.gscloud.cn/, accessed on 30 June 2025). The sampling point is at an altitude of 2696.60 m. The climate in this region is extremely arid, and the soil is severely salinized [20,21].

Soil samples were collected by the ring knife method [15,22]. Select 3 individuals of *A. sinodeliciosus* and *C. comatus*, respectively. Use the ring knife (100 cm^3^, 50.4 mm × 50 mm) to collect soil sample beneath each fruiting body, providing 3 biological replicates of soil samples for *A. sinodeliciosus* and *C. comatus*. Similarly, using the ring knife, collect 3 soil samples from the midpoint between the two species of macrofungi growth areas as the control. In China, *A. sinodeliciosus* is called “Da fei gu”, so the soil is marked as “DFG”. *C. comatus* is called “Ji tui gu”, and the soil is labeled as “JT”. The control soil is marked as “WT”. All the soil samples were divided into two parts. One part was used for physical and chemical property detection, and the other part was quickly frozen in liquid nitrogen and placed in dry ice, then sent to BGI for metagenomic sequencing. Subsequently, the three biological replicates of WT, DFG, and JT were analyzed individually.

### 2.2. Determination of Soil Physiochemical Properties

The soil water content was measured by drying method. After indoor air drying and impurity removal, the soil samples were ground through a 2 mm mesh screen, and mixtures were prepared with distilled water at weight-to-volume ratios of 1:2.5 and 1:5, respectively, for pH and electrical conductivity measurements [23]. Refer to the method reported by Jones and Willett [24], determine soil total carbon (TC), total nitrogen (TN) and hydrolyzable nitrogen (HN) contents were measured using an Elemental analyser (VarioEL III, Hanau, Germany). Soil available phosphorus (AP) was assayed using a continuous flow analytical system (SKALAR SANþþ, Breda, The Netherlands) [23], and available potassium (AK) and total salt were quantified using atomic emission spectrometry (ICPS-7500, Shimadzu, Osaka, Japan) [25].

### 2.3. Metagenomic Data Analysis

#### 2.3.1. Data Assembly

Genomic DNA was extracted from 0.25 g of soil samples using the DNeasy PowerSoil Pro Kit (Qiagen, Venlo, The Netherlands) according to the manufacturer’s protocol, incorporating steps of precipitation, silica membrane adsorption, and multiple wash steps for purification [26]. The resulting DNA was subjected to quality control via Nanodrop spectrophotometry, Qubit fluorometry (Thermo Fisher Scientific, Wilmington, NC, USA), and electrophoretic analysis. Library construction involved DNA fragmentation, adapter ligation, and PCR amplification, followed by final library quality assessment prior to sequencing. Libraries were sequenced on the DNBSEQ-T7 platform using a paired-end 150 (PE150) strategy, generating 150-bp reads and achieving a sequencing depth of 10 Gb per sample. The raw sequencing data have been deposited in the NCBI Sequence Read Archive (SRA) under accession number SUB15753403.

The sequencing raw data were quality-trimmed and filtered using the software Trimmomatic (version 0.33) to obtain high-quality reads. The metagenome assembly was conducted using the splicing software Megahit (version 1.1.2). The assembly was performed using a sample pooling strategy, where all sample sequences were first combined and then assembled using the default parameters of megahit. Open reading frames (ORFs) of the assembled contigs were predicted using Prodigal (version 2.6.3) software. CD-HIT (version 4.7) (http://www.bioinformatics.org/cd-hit/,accessed on 8 October 2023) was used to cluster the predicted sequences of genes (identity > 95%, coverage > 90%), the redundant genes in the longest gene sequence were removed to construct the corresponding non-redundant gene set [15]. Gene quantification was performed using “kallisto” (version 0.45.1) software to obtain the expression levels of each non-redundant gene in each sample.

#### 2.3.2. Analysis of Microbial Composition

Species annotation was performed by aligning with the Kraken2 and the self-built microbial nucleic acid database to calculate the number of sequences of the species in the sample [27]. The microbial relative abundance was calculated using Bracken (version 2.9) software [28]. The processed data was uploaded to Pavian (shinyapps.io) to obtain a microbial composition Sanping diagram, and then the microbial species composition and species abundance information statistics were performed using the braken (version 2.9) software, and the abundance spectrum was refined to the domain, kingdom, phylum, class, order, family genus, and species taxonomic levels [26].

#### 2.3.3. Analysis of Microbial Diversity

Three alpha-diversity indices—Shannon, Simpson, and Pielou’s evenness—were computed using the vegan package (v2.7-2) in R (v4.3.2) [29], with a significance level set at *p* < 0.05. The Shannon index assessed overall microbial diversity, the Simpson index reflected dominance characteristics, and Pielou’s index quantified species distribution uniformity. Shannon and Simpson indices were generated via the functions diversity(otu_table, index = “shannon”) and diversity(otu_table, index = “simpson”), respectively. Pielou’s index was derived as J′ = H′/ln(S), where H′ represents the Shannon index and S denotes observed species richness [30]. To mitigate sequencing depth variation, all samples were subjected to rarefaction using the rarefy() function from vegan, standardizing read counts across samples and minimizing depth-related bias. This normalization ensured robust and comparable diversity estimates throughout the analysis. Principal coordinate analysis (PCoA) and Bray–Curtis Anosim analysis were conducted to describe the beta diversity of soil microorganisms [31].

#### 2.3.4. Key Differential Microorganism Discovery

The LEfSe (version 1.1.2) software was used to analyze the differences in key microorganisms between groups [27]. First, a clustering analysis of the samples was conducted to generate an OTU table. The OTU tables from each group were then organized into an OTU matrix. Using the LEfSe software, the matrix was analyzed to identify species with significant differences between groups. Subsequently, linear discriminant analysis (LDA) was employed to assess the correlation between the abundance of each species and the differences observed in each group, with a threshold set at 3.0.

#### 2.3.5. Analysis of Microbial Symbiotic Networks

The Pearson correlation between species was calculated using the psych in R language, and the data with absolute value of correlation coefficient r greater than 0.8 and *p* value less than 0.05 were selected to the network file (graphml format file in the corresponding path of the Network folder), and the visualization was performed using Gephi (version 1.0) and Cytoscape (version 3.6.1) [32]. In network topology analysis, degree centrality and clustering coefficient serve as key metrics for characterizing interspecies relationships. Degree quantifies the number of direct connections per node (species), where elevated values indicate ecologically pivotal taxa within the community. The clustering coefficient measures local interconnectivity density; higher values reflect tightly knit interaction modules that may enhance ecological stability and facilitate cooperative dynamics.

#### 2.3.6. Statistical Analyses

Using SPSS 22 software, one-way ANOVA and the least significant difference (LSD) method were employed to compare the significant differences in the physicochemical properties of soil samples and the relative abundance of microorganisms among groups, with a significance level set at *p* < 0.05. The relationship between microorganisms and environmental factors was analyzed using the Mantel test and redundancy analysis (RDA) in the “vegan” package of R software (version 2.5.3) [30].

#### 2.3.7. AI-Assisted Language and Structural Polishing

In Section 4, we used the large language model Kimi (developed by Moonshot AI) to assist with language polishing, structural optimization, and idea organization. The AI was not involved in data analysis, experimental design, or the interpretation of results. All AI-generated content was carefully reviewed, revised, and fact-checked by the authors to ensure academic accuracy and logical consistency.

## 3. Results

### 3.1. The Effects of A. sinodeliciosus and C. comatus on Soil Physicochemical Properties

The results of the soil physicochemical property analyses for each group are shown in Table 1. Compared with the control group, the soil electrical conductivity (EC) of *A. sinodeliciosus* and *C. comatus* decreased by 98.63% and 99.67%, respectively. Meanwhile, the total carbon (TC) content increased by 27.48% and 113.24%, total nitrogen (TN) by 95.16% and 108.06%, hydrolyzable nitrogen (HN) by 87.36% and 97.90%, available potassium (AK) by 182.72% and 596.09%, and total salt content by 675.28% and 109.69%, respectively. The results demonstrate that both macrofungi species can enhance soil nutrient indices and improve soil fertility, with *C. comatus* exhibiting a stronger fertilization effect. Meanwhile, while both fungi increased the total salt content in the surrounding soil, they led to a reduction in soil EC. In addition, *C. comatus* markedly increased available phosphorus (AP) by 163.14%, whereas *A. sinodeliciosus* had no significant effect on AP.

We observed a substantial increase in AK content in both the DFG and JT groups. The increase in AK indicates enhanced transformation of potassium from mineral or fixed pools into plant- and microbially available forms. This serves as a key indicator of active nutrient cycling and is directly and indirectly linked to fungal and bacterial activities. The decline in electrical conductivity (EC) typically reflects a reduction in the total concentration of soluble ions in the soil solution. A decrease in EC suggests shifts in microbial immobilization and resource allocation.

**Table 1:** Physicochemical properties of soil samples.

Physicochemical Properties	WT	DFG	JT
Water content (W, %)	5.71 ± 0.76 a	7.92 ± 0.62 b	6.38 ± 0.15 a
Electrical conductivity (E, µS/cm)	1638.67 ± 145.65 a	22.43 ± 0.81 b	5.45 ± 0.54 b
pH	7.93 ± 0.55 a	8.75 ± 0.26 b	7.62 ± 0.93 c
Total carbon (TC, g/kg)	25.22 ± 1.20 a	32.15 ± 2.28 b	53.78 ± 1.44 c
Total nitrogen (TN, g/kg)	1.86 ± 0.21 a	3.63 ± 0.14 b	3.87 ± 0.17 b
Hydrolyzable nitrogen (HN, mg/kg)	162.31 ± 30.02 a	304.10 ± 19.77 b	321.21 ± 11.93 b
Available phosphorus (AP, mg/kg)	8.22 ± 1.49 a	8.80 ± 1.64 a	21.63 ± 2.00 b
Available potassium (AK, mg/kg)	382.02 ± 9.35 a	1080.02 ± 32.60 b	2659.19 ± 112.87 c
Total salt (TSalt, g/kg)	16.30 ± 3.04 a	126.37 ± 2.74 b	34.18 ± 11.37 c

WT, soil of the control; DFG, soil of *A. sinodeliciosus;* JT, soil of *C. comatus*. Data are presented as mean ± standard deviation (SD). Different letters represent significant differences (*p* < 0.05).

### 3.2. Effects of A. sinodeliciosus and C. comatus on Soil Microorganisms

#### 3.2.1. Effects on Soil Microbial Content

The microbial content of the soil samples is shown in Table 2. Compared with the control, *A. sinodeliciosus* increased total microbial content by 24.80%, bacterial content by 25.91%, and fungal content by 550%. In the soil of *C. comatus*, total microbial content and bacterial content rose by 31.62% and 31.77%, respectively, whereas fungal content showed no significant change.

#### 3.2.2. Effects on Soil Microbial Composition

**Figure 2:**
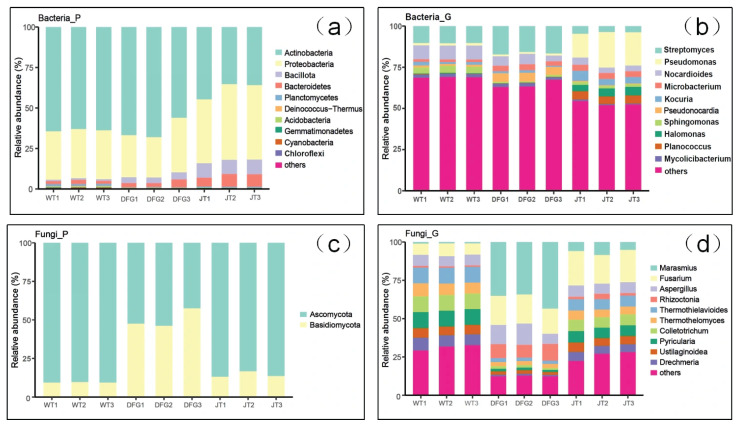
Distribution of microbial abundance in soil samples. (**a**), distribution of soil bacteria at the phylum level; (**b**), distribution of soil bacteria at the genus level; (**c**), distribution of soil fungi at the phylum level; (**d**), distribution of soil fungi at the genus level. WT, soil of the control; DFG, soil of *A. sinodeliciosus*; JT, soil of *C. comatus*.

The distribution of microbial abundance in the soil samples is shown in Figure 2. Combined one-way ANOVA and least significant difference (LSD) tests (*p* < 0.05) revealed that: At the phylum level of bacteria, compared with WT, *A. sinodeliciosus* exerted no significant impact on the dominant bacterial phyla in the soil, but significantly elevated the relative abundance of *Bacillota*. *C. comatus* greatly reduced the abundance of *Actinobacteria* and increased the abundance of *Proteobacteria*, making *Proteobacteria* become the dominant phylum. At the genus level of bacteria, *A. sinodeliciosus* significantly increased the relative abundance of *Streptomyces*, promoting it to a dominant genus. *C. comatus* significantly decreased the relative abundance of *Streptomyces* while markedly increasing that of *Pseudomonas*, thereby promoting *Pseudomonas* to a dominant genus.

At the phylum level of fungi, *A. sinodeliciosus* significantly reduced the abundance of *Ascomycota* and increased that of *Basidiobolomycota*, making the latter the dominant fungal phylum. At the genus level, *A. sinodeliciosus* greatly increased the relative abundance of *Marasmius*, *Fusarium*, and *Rhizoctonia.* At both the phylum and genus levels, *C. comatus* showed no substantial effect on the relative abundance of soil fungi.

#### 3.2.3. Effects on Soil Microbial Diversity

(1) Alpha diversity analysis

**Figure 3:**
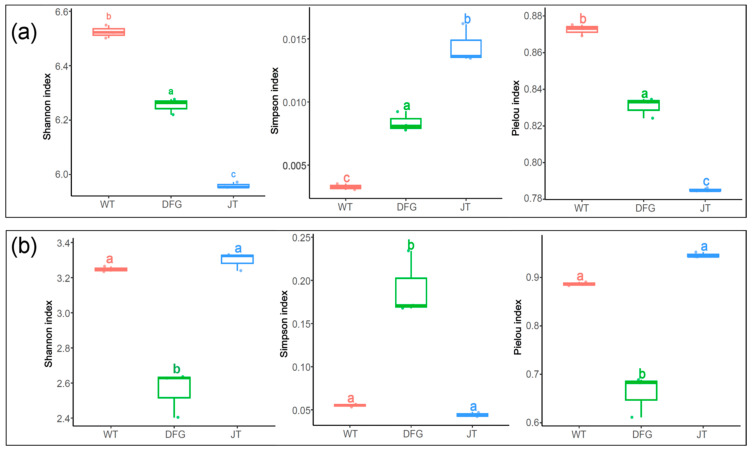
Alpha diversity analysis of soil microorganism based on species level. (**a**), bacteria; (**b**), fungi. WT, soil of the control; DFG, soil of *A. sinodeliciosus*; JT, soil of *C. comatus.* Different letters represent significant differences (*p* < 0.05).

The impacts of *A. sinodeliciosus* and *C. comatus* on soil microbial diversity are presented in Figure 3. Both macrofungi significantly decreased the alpha diversity of soil bacteria. Among them, *C. comatus* led to a greater reduction. Concerning fungi, *A. sinodeliciosus* reduced the alpha diversity, whereas *C. comatus* had no significant influence on the alpha diversity of fungi.

(2) Beta diversity analysis

**Figure 4:**
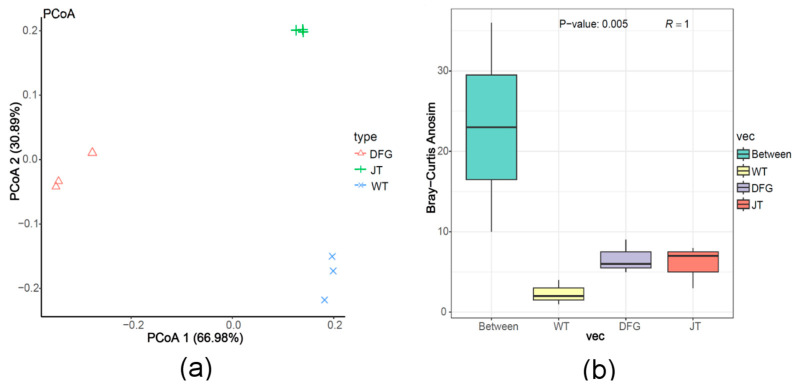
Beta diversity analysis of soil microorganism. (**a**), PCoA analysis; (**b**), Anoism analysis. WT, soil of the control; DFG, soil of *A. sinodeliciosus*; JT, soil of *C. comatus*.

Beta diversity, also known as between-habitat diversity, is often used to compare whether there are differences between different samples. The PCoA analysis results are shown in Figure 4a, where samples in the same group are close to each other, and samples in different groups are clearly separated. This indicates that microbial community similarity of the same group of samples is high, the samples are relatively uniform, and there are some differences in the microbial community between different groups of samples. Further Anoism significance analysis (Figure 4b) showed the R value was 1, indicating that the difference between groups was much greater than the difference within groups. The *p*-value was 0.005 (less than 0.05), indicating that the statistics were significant, that is, there were significant differences in microbial communities among WT group, DFG group and JT group, and beta diversity was high. This further proved that the growth of *A. sinodeliciosus* and *C. comatus* had a significant impact on the structure of soil microbial communities.

#### 3.2.4. Marker Microbial Analysis

**Figure 5:**
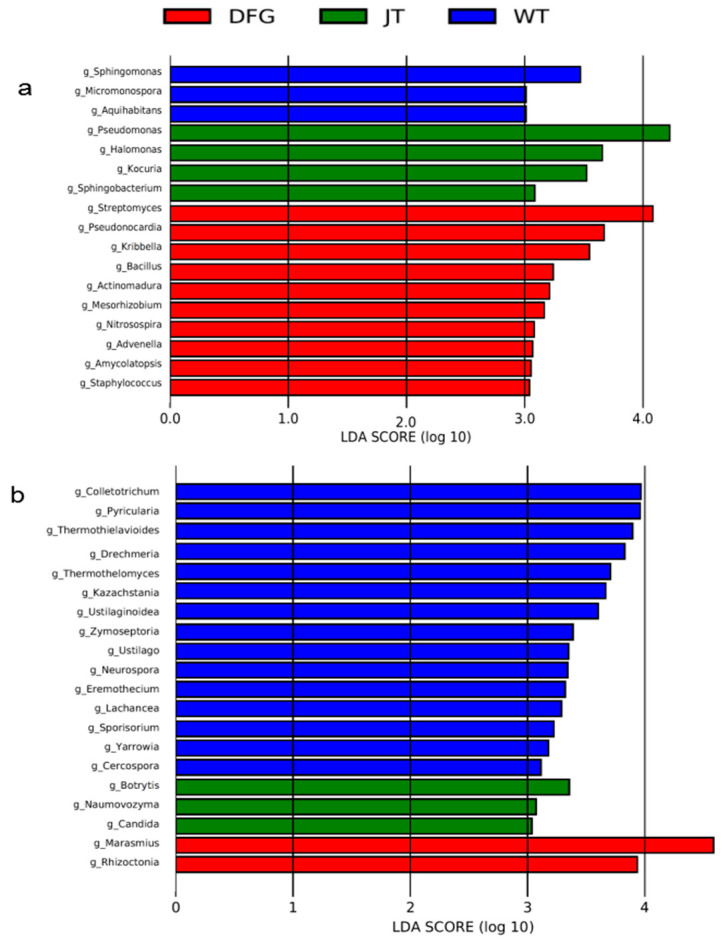
LDA bar chart of inter-group LEfSe analysis based on relative abundance of species. (**a**): bacteria; (**b**): fungi. WT, soil of the control; DFG, soil of *A. sinodeliciosus*; JT, soil of *C. comatus*.

To understand the key microorganisms leading to the differences among the various groups of soil samples, a biomarker analysis was conducted by means of LEfSe software, and the intergroup differential microorganisms were sorted according to the LDA value. The analysis results are shown in Figure 5. At the genus, there were significant differences in the bacterial biomarkers of each group, with a total of 17 genera (3 in the WT group, 4 in the JT group, and 10 in the DFG group) that had LDA values greater than 3.0. Among them, *Pseudomonas* in the JT group and *Streptomyces* in the DFG group had LDA values greater than 4.

Fungal markers at the genus level in each group are shown in Figure 5b. Compared with WT, there were 3 fungal markers that were different in JT group, including *Botrytis*, *Naumovozyma*, and *Candida*. The DFG group had two different fungal markers, *Marasmius* and *Rhizoctonia*, among which the LDA value of *Marasmius* was higher than 4.0.

### 3.3. Soil Microbial Correlation Analysis

**Figure 6:**
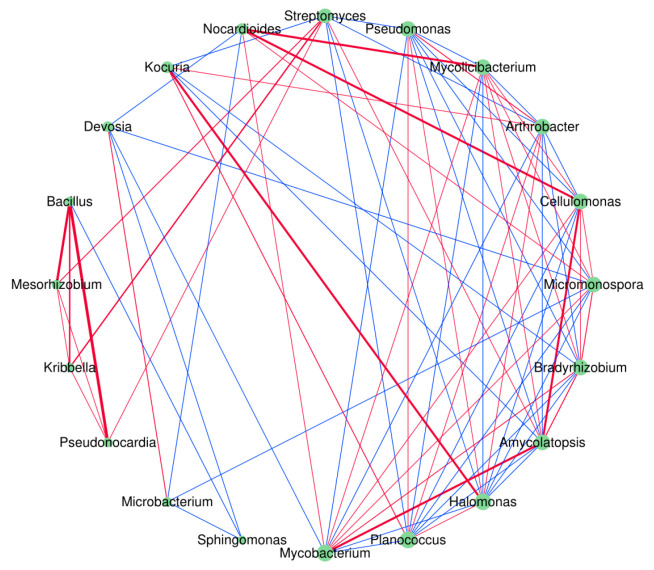
Correlation analysis of the top 20 most abundant microorganisms. The red lines indicate positive correlations, while the blue lines represent negative correlations; the thicker the line, the stronger the correlation.

Correlation analysis was performed on the top 20 microorganisms in abundance among the three groups of samples at the genus level (Figure 6). In this study, co-occurrence networks were constructed for both bacterial and fungal communities. However, due to the limited interaction density observed in the fungal network, all subsequent topological analyses were exclusively performed on the bacterial network, with no further examination of fungal interactions.

It can be seen that there is a certain negative correlation between *Streptomyces* and *Pseudomonas*. In addition, *Nocardioides* and other 4 genera, such as *Mycolicibacterium*, have a certain positive correlation, and negative correlation with *Microbacterium* and *Devosia*. *Mycobacterium* has a positive correlation with 6 genera, such as *Amycolatopsis*, and certain negative correlation with *Arthrobacter*, *Pseudomonas*, and *Devosia*. *Cellulomonas* has a positive correlation with 6 genera, such as *Amycolatopsis* and *Nocardioides*, and a negative correlation with 4 genera including *Arthrobacter*. This shows that the microorganisms in the soil of *A. sinodeliciosus* and *C. comatus* have close and complex interactions, either promoting each other or inhibiting each other, to maintain the normal life activities of the two macrofungi.

### 3.4. Correlation Analysis Between Soil Microorganisms and Physicochemical Properties

**Figure 7:**
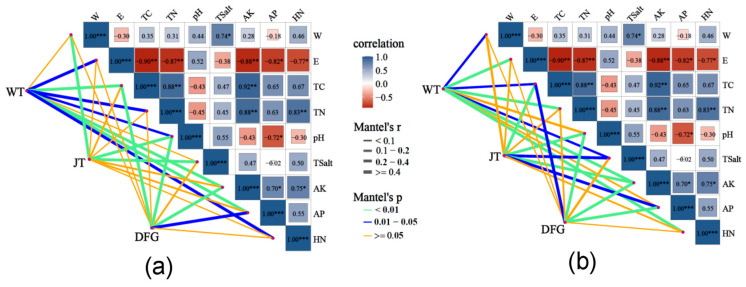
Correlation analysis between microbial abundance and physicochemical properties in soil. (**a**), bacteria; (**b**), fungi. WT, soil of the control; DFG, soil of *A. sinodeliciosus*; JT, soil of *C. comatus*. *p* < 0.05 (*), *p* < 0.01 (**), *p* < 0.001 (***).

The correlation analysis of soil bacteria and fungi with physicochemical properties was carried out, respectively, and the results are shown in Figure 7. Spearman correlation analysis indicated that nutritional indicators such as TC, TN, AK, AP, and HN were all significantly negatively correlated with electrical conductivity. TC, TN, and AK were significantly positively correlated with each other, and TN was also significantly positively correlated with HN. The Mantel test results showed that in terms of bacterial abundance, the bacterial abundance in the WT group was extremely significantly correlated with TC, AK, and AP, and significantly correlated with electrical conductivity, TN, pH, and HN. In the JT group, bacterial abundance was extremely significantly correlated with water content, pH, and TSalt. In the DFG group, bacterial abundance was extremely significantly correlated with TC, pH, and AK. In terms of fungal abundance, the fungal abundance in the WT group was extremely significantly correlated with electrical conductivity, TN, AK, and HN, and significantly correlated with water content, TC, and TSalt. In the JT group, fungal abundance was extremely significantly correlated with TC, pH, and AK, and significantly correlated with TSalt and AP. In the DFG group, fungal abundance was extremely significantly correlated with pH, AK, and AP, and significantly correlated with TC.

**Figure 8:**
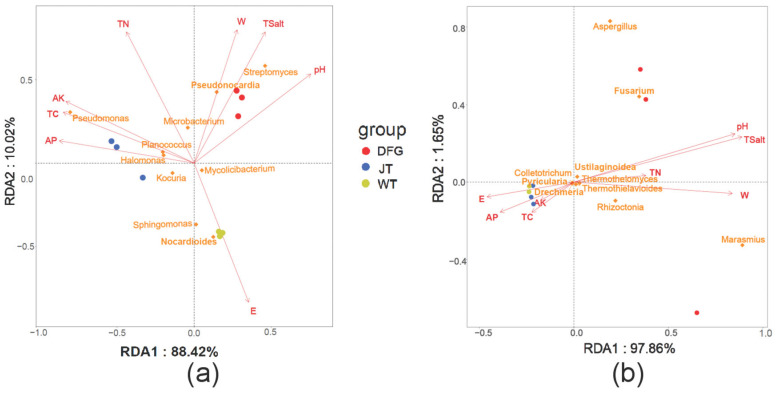
Correlation analysis between the top ten microbial genera and physicochemical properties. (**a**), bacteria; (**b**), Fungi. WT, soil of the control; DFG, soil of *A. sinodeliciosus*; JT, soil of *C. comatus*.

To further clarify the relationship between soil microbial abundance and physicochemical properties, taking soil physicochemical properties as explanatory variables and the abundance of the top ten bacterial and fungal genera in relative abundance as response variables, redundancy analysis (RDA) was conducted on the physicochemical property factors and soil microorganisms. As can be seen from Figure 8a, based on the projection lengths of different soil physical and chemical factors on the first axis, pH, AP, TC, and AK are the most significant factors influencing bacterial composition. TN, Tsalt, electrical conductivity, and water content are also important factors. The RDA results show that pH, Tsalt, and water content are positively correlated with *Streptomyces*, *Pseudonocardia*, and *Microbacterium*, and negatively correlated with *Pseudomonas*, *Nocardioides*, *Sphingomonas*, and *Kocuria*, etc. TC, TN, AP, and AK are positively correlated with *Pseudomonas*, *Planococcus*, *Halomonas*, and *Microbacterium*, etc., and negatively correlated with *Nocardioides*, *Sphingomonas*, and *Streptomyces*, etc. The study on the correlation between electrical conductivity and TN is just the opposite. Figure 8b shows that total salt is the most important factor affecting fungi. *Marasmius*, *Fusarium*, *Aspergillus*, and *Rhizoctonia* are positively correlated with total salt, pH, moisture content, and TN, and negatively correlated with electrical conductivity, AP, TC, and AK.

## 4. Discussion

This study, through the combination of metagenomic sequencing and soil physicochemical analysis, systematically evaluated the reshaping effects of two precious macrofungi, *A. sinodeliciosus* and *C. comatus*, on the rhizosphere soil microecosystem in the saline-alkali land of the Qaidam Basin, Qinghai Province, China. The research found that the two macrofungi not only significantly improved the soil nutrient status but also formed a unique “fungus-bacteria-soil” interaction network by selectively regulating the microbial community structure.

### 4.1. Macrofungi Enhance Soil Fertility

Both *A. sinodeliciosus* and *C. comatus* significantly increased key soil nutrient indicators such as TC, TN, HN, and AK, with *C. comatus* showing a more prominent fertilization effect (Table 1). Reports of macrofungi enhancing soil fertility have also been observed in previous studies [33,34]. Rashid et al. [35] found that cultivating *Morchella esculenta* under forests could promote cooperation between fungi and bacteria, enhancing soil nutrients. Hu et al. [33] discovered that *Stropharia rugosoannulata* in rotation with rice could significantly increase soil organic carbon content (30.2–31.2%) and rice yield (16.0–17.0%). Cultivating *S. rugosoannulata* under mountain forests significantly increased soil nutrient accumulation, with significant increases in soil organic carbon, TC, TN, AP, and the activities of glucosidase and cellulase [36]. Medina et al. [34] confirmed that *A. bisporus* mushroom residue could significantly increase soil oxidizable organic carbon, organic nitrogen, and AP levels. Similarly, *Pleurotus ostreatus* releases carbon and nitrogen by degrading lignocellulose, directly promoting soil organic matter accumulation [37]. These results collectively indicate that the enhancement of soil nutrients by macrofungi is universal across species and ecosystems, and the mechanism mainly relies on the secretion of extracellular enzymes by mycelia to decompose complex organic matter [38].

*Agarious* (e.g., *A. bisporus*) secretes manganese peroxidase, laccase, and lignin peroxidase to degrade complex lignocellulose, significantly altering the composition of the soil carbon pool and influencing soil microbial communities [39]. Coprinus species rapidly utilize simple carbon sources, contribute to microbial necromass carbon, and interact with other microorganisms to generate microecological effects [38].

Macrofungi degrade lignocellulose by secreting extracellular cellulases, hemicellulases and ligninolytic enzymes such as laccases, thereby releasing soluble carbon that fosters humus formation and increases soil organic-C content and porosity [40]. The resulting low-molecular-weight carbon compounds stimulate the colonization of nitrifying and denitrifying communities and accelerate the mineralization–immobilization turnover of nitrogen [41]. Simultaneously, organic acids and acid phosphatases exuded by the fungi convert fixed phosphorus into soluble phosphate, directly enhancing soil P supply [40]. Through these potential functional pathways, macrofungi participate in the soil C–N–P coupling that regulates both nutrient status and physico-chemical properties [41].

### 4.2. Macrofungi Reshape Soil Microbial Communities

In terms of soil microbial composition, *A. sinodeliciosus* increased the relative abundance of *Streptomyces* bacteria, making them the dominant genus (Figure 2), which is consistent with the research results of Xing et al. [15]. *Streptomyces*, as important antibiotic-producing genus [42], may create a healthy rhizosphere environment for *A. sinodeliciosus* by inhibiting pathogenic bacteria. *C. comatus* significantly reduced the relative abundance of *Streptomyces* and increased the relative abundance of *Pseudomonas* (Figure 2). LEfSe (Figure 5) and correlation analysis (Figure 6) revealed that *Streptomyces* and *Pseudomonas* were significantly negatively correlated. The negative correlation suggests direct antagonism, likely through antibiotic production by Streptomyces [43]. *Streptomyces* promotes carbon and nitrogen cycling while suppressing soil-borne diseases. It may shape fungal community composition through antagonistic interactions [44]. Meanwhile, core microbial groups such as *Nocardioides*-*Mycolicibacterium* formed positive correlation clusters, jointly maintaining the stability of the mycorrhizosphere microecology. This microecological effect has also been found in the cultivation process of *A. bisporus* [45]. In terms of soil fungi, *A. sinodeliciosus* decreased the relative abundance of *Ascomycota* and increased the abundance of *Basidiobolomycota* (Figure 2), while *C. comatus* had no significant effect on the composition of soil fungi. In recent years, research on the impact of macrofungi on soil microorganisms has been increasing. Ke et al. [46] found that the composition of soil microorganisms changed during the cultivation experiment of the medicinal mushroom *Ganoderma lingzhi*, and the fungal community showed greater changes than the bacterial community in both species richness and relative abundance of several dominant species. Cai et al. [47] found that there was a mutualistic relationship between mushroom mycelium growth and soil bacteria, with an increase in bacterial numbers accompanying mushroom growth. Vaario et al. [48] found *Tricholoma matsutake* coexists with a variety of fungi and actinobacteria.

In terms of microbial diversity, *A. sinodeliciosus* significantly reduced the α diversity of both bacteria and fungi, while *C. comatus* reduced the α diversity of bacteria but had no significant effect on the α diversity of fungi (Figure 3). Similarly, McGee et al. [39] found that *A. bisporus* led to a decrease in soil microbial diversity. Feng et al. [49] reported that the cultivation of Morchella esculenta in soil significantly reduced microbial α diversity.

### 4.3. Coupling Relationship Between Microorganisms and Physicochemical Properties

The results of RDA and Mantel tests (Figure 7 and Figure 8) indicate that bacterial communities are mainly driven by factors such as pH, TC, and AK, while fungal communities are more susceptible to total salt (Tsalt). This finding supports the “microbial niche differentiation” theory, which posits that differences in response thresholds to environmental factors among different groups lead to community structure differentiation [50]. Notably, the AP content in the soil of *C. comatus* was significantly higher than that of *A. sinodeliciosus* (Table 1), and the RDA results showed a positive correlation between AP and *Pseudomonas* (Figure 8a), which was significantly enriched by *C. comatus* and the marker microorganism for it. This result aligns with previous reports that *Pseudomonas* possesses phosphate-solubilizing capabilities [51,52]. Additionally, *Pseudomonas* also has plant growth-promoting, such as nitrogen fixation and siderophore production, and stress-tolerance characteristics, such as salt and alkali tolerance [52], which helps explain the stronger soil fertility improvement ability of *C. comatus* compared to *A. sinodeliciosus* and its adaptation to the saline-alkali environment of the Qaidam Basin. Moreover, the electrical conductivity was negatively correlated with most nutrient indicators (Figure 7), suggesting that salt stress may inhibit microbial activity through osmotic pressure effects, while macrofungi may alleviate this inhibition by improving soil aggregate structure [35]. Meanwhile, while both fungi increased the total salt content in the surrounding soil, they led to a reduction in soil electrical conductivity (Table 1), suggesting biological sequestration. Salts may be incorporated into fungal and microbial biomass or complexed with newly produced organic compounds, reducing their ionic form in the soil solution and thus the electrical conductivity [53,54]. These results all indicate that *A. sinodeliciosus* and *C. comatus* affect the physicochemical properties and improve soil nutrient conditions by selectively regulating microbial community structure, forming a unique “fungus–bacteria–soil” interaction network.

Currently, studies on “fungus–bacteria–soil” interactions are not uncommon [55]. For instance, cellulose-decomposing fungi, including Ascomycetes and Basidiomycetes, play a crucial role in degrading the main polysaccharide cellulose in soil, which is beneficial for mycelial growth and mushroom yield [39]. The research results of Zhou et al. [6] are similar to this view and also confirm that some *Bacillota* or *Ascomycota* microorganisms may have the ability to increase mushroom yield. Additionally, Rashid et al. [35] demonstrated that cultivating *Morchella esculenta* under forest can promote the cooperation between fungi and bacteria and enhance soil nutrients. The increase in the relative abundance of *Bacillota* can drive the improvement of soil AP [56]. Similarly, Li et al. [57] found in the soil of vegetation restoration in red soil regions that soil pH, available phosphorus content, and available potassium content were positively correlated with the relative abundance of *Chloroflexi*. In addition, *Arbuscular mycorrhizal* fungi act as keystone taxa in the revegetation of the Tibetan Plateau [58]. Synergistic effects within the fungal community promote the growth and yield of oilseed rape; in particular, *Alternaria* strains isolated from rape tissues significantly enhance seed germination of Brassica rapa under low-temperature and drought stress [59].

## 5. Conclusions

Macrofungi play a crucial role in terrestrial ecosystems as important decomposers, influencing soil nutrient cycling and microbial growth. We found that both *A. sinodeliciosus* and *C. comatus* significantly increased soil nutrient indicators such as TC (27.48% and 113.24%, respectively), TN (95.16% and 108.06%, respectively), HN (87.36% and 97.90%, respectively), and AK (182.72% and 596.09%, respectively), enhancing soil fertility. Moreover, *C. comatus* had a stronger ability to improve soil fertility. At the same time, both macrofungi also affected the microbial community of the soil. *A. sinodeliciosus* significantly increased the relative abundance of *Streptomyces* and *Marasmius*, and decreased the α diversity of soil bacteria and fungi. *C. comatus* significantly increased the relative abundance of *Pseudomonas* and decreased the α diversity of bacteria, but had no significant effect on the composition and diversity of fungi. There is a complex interrelationship between the physicochemical properties and the microbial community. pH, AP, TC, and AK are the main factors affecting bacteria, while total salt is the main factor affecting fungi. However, the interaction mechanism between the microorganisms and physicochemical properties of the soil adjacent to *A. sinodeliciosus* and *C. comatus* awaits further study.

## Figures and Tables

**Table 2:** Microbial content of soil samples.

Item	WT	DFG	JT
Effective sequence (reads)	40,100,457.67 ± 24,815.72 a	40,075,380.67 ± 56,909.47 a	40,092,891.33 ± 12,338.52 a
Effective sequence (%)	28.87 ± 0.31 a	36.03 ± 1.62 b	38.03 ± 0.45 b
Unknown species sequences (%)	71.13 ± 0.31 a	63.97 ± 1.62 b	61.97 ± 0.45 b
Microbial sequences (%)	28.87 ± 0.31 a	36.03 ± 1.62 b	38.00 ± 0.4 b
Bacterial sequence (%)	28.33 ± 0.25 a	35.67 ± 1.63 b	37.33 ± 0.45 b
Fungal sequence (%)	0.02 ± 0.06 a	0.13 ± 0.02 b	0.02 ± 0.06 a
viral sequence (%)	0.01 ± 0.00 a	0.03 ± 0.01 b	0.01 ± 0.00 a

WT, soil of the control; DFG, soil of *A. sinodeliciosus*; JT, soil of *C. comatus*. Data are presented as mean ± standard deviation (SD). Different letters represent significant differences (*p* < 0.05).

## Data Availability

Restrictions apply to the availability of these data. Data were obtained from NCBI and are available https://www.ncbi.nlm.nih.gov/ with the permission of NCBI. accession number SUB15753403, accessed on 14 November 2025.

## Abbreviations

The following abbreviations are used in this manuscript:

DFG	*Agaricus sinodeliciosus*, called “Da Fei Gu” in China
JT	*Coprinus comatus*, called “Ji Tui Gu” in China
WT	The control
TC	Total carbon
TN	Total nitrogen
HN	Hydrolyzable nitrogen
AP	Available phosphorus
AK	Available potassium
TSalt	Total salt
